# Electrochemical Degradation of Tetracycline Using a Ti/Ta_2_O_5_-IrO_2_ Anode: Performance, Kinetics, and Degradation Mechanism

**DOI:** 10.3390/ma14154325

**Published:** 2021-08-02

**Authors:** Hao Dong, Wanqiang Chi, Ang Gao, Tianyu Xie, Bo Gao

**Affiliations:** 1 School of Environmental Science and Engineering, Qilu University of Technology (Shandong Academy of Sciences), Jinan 250353, China; 17854118848@163.com (H.D.); ty932950009@163.com (T.X.); 2 Ecological Environment Bureau of Weihai City (Rongcheng Branch Office), Weihai 264300, China; recp@163.com; 3 School of Civil Engineering and Architecture, Wuhan University of Technology, Wuhan 430070, China; gao15169151617@163.com; 4 School of Energy and Power Engineering, Qilu University of Technology (Shandong Academy of Sciences), Jinan 250353, China

**Keywords:** antibiotics, tetracycline, DSA, Ti/Ta_2_O_5_-IrO_2_ anode, electrochemical degradation, degradation mechanism

## Abstract

Tetracycline (TC) is widely used in production and in life. The high volume of its use and the difficulty of its disposal have become the most important causes of environmental pollution. A suitable method needs to be found to solve this problem. In this study, the Ti/Ta_2_O_5_-IrO_2_ electrode was characterized for its surface morphology and crystal composition. The electrochemical catalytic ability of the Ti/Ta_2_O_5_-IrO_2_ electrode was investigated using LSV and CV tests. The electrochemical degradation of tetracycline (TC) in water with a Ti/Ta_2_O_5_-IrO_2_ anode was investigated. The main influence factors, such as current density (2.5–10 mA/cm^2^), electrode spacing (20–40 mm), initial TC concentration (20–80 mg/L) and initial solution pH (4.74–9.48) were analyzed in detail and their influences on reaction kinetics was summed up. The removal rate increased along with the increasing current density, decreasing initial TC concentration and decreasing of electrode distance under the experimental conditions. The optimum pH was 4.74. UV–vis, total organic carbon (TOC) and high-performance liquid chromatography-mass spectrometry (HPLC-MS) analyses were used to reveal the mechanism of TC degradation. Nine main intermediates were identified, and the degradation pathways were proposed. A new insight has been postulated for the safe and efficient degradation of TC using the Ti/Ta_2_O_5_-IrO_2_ electrode.

## 1. Introduction

Antibiotics are widely used for disease treatment in humans and as prophylaxis and growth promoters for livestock [1]. Because of poor adsorption in the gut of animals, as much as 90% of used antibiotics are excreted in the urine, and 75% remains unchanged in the feces [2,3]. Tetracycline (TC), a kind of broad-spectrum antibiotic, is widely used as a veterinary pharmaceutical across the world. Studies have shown that conventional biological wastewater treatment systems were only moderately effective in the removal of antibiotics [4,5] The occurrences of TC have been reported in fresh water [6,7], groundwater [8], soil [9,10] and sediment [10,11,12] which may pose serious risks to both human and animal health. Thus, it is necessary to investigate physical–chemical treatment technologies to further improve the removal of TC.

Electrochemical oxidation is a kind of advanced oxidation process, during which refractory organic compounds may be effectively oxidized or mineralized under the action of an electric field and can effectively avoid secondary pollution. This technology has the advantage that the entire degradation process can be easily controlled and does not require the addition of other redox agents. It is considered “environmentally friendly” [11,13,14] and has been widely used in the degradation of antibiotics [12,15,16]. Electrode plays a key role during the electrochemical process. The electrode reaction occurs between the electrode and the solution interface [13]. When the electrode is in contact with the solution to form a new interface, the free charge from the liquid phase will rearrange on the interface to form a double electric layer. When the power is turned on, the electrons on the electrode that can move freely can form a double electric layer with various particles in the solution. Once the electrode surface has enough charge to reach the electrode potential for the redox reaction of particles or molecules, the redox reaction will occur in the aqueous phase [14,15]. Thus, choosing appropriate electrode materials is an effective way to improve the efficiency of the electrochemical catalytic reaction.

Dimensionally stable anode (DSA), developed in the late 1960s and early 1970s, is a kind of anode made by coating metal oxide on a titanium plate. Due to the excellent performance of DSA electrodes, they have been extensively used in the degradation of refractory compounds [16,17,18]. However, due to the weak bonding between the active coating and the substrate, the peeling phenomenon of the active coating is easy to occur, which leads to the decrease in the catalytic activity and the passivation of the substrate. Many researches have shown that the doping of nonmetal, transition metal or both in the coating can effectively improve the catalytic activity of the DSA electrode and can improve the structural defects of the coating. Wang [12] used a Ti/Ti_4_O_7_ electrode to electrochemically degrade TC. The results showed that the degradation rate of 20 mg/L of TC in 40 min was 90.60%. Yang [17] used the SnO_2_–Sb–Ce/Ti electrodes to degrade 10 mg/L of TC with a degradation rate of 72.4%. Frontistis [18] prepared a BDD electrode and catalyzed the degradation of TC. The results showed that the prepared BDD electrode had better efficiency than a platinum electrode and a stainless-steel electrode, which increased the TC degradation effect by 20%. Brinzila [19] used the BDD electrodes to electrochemically degrade tetracycline. Studies have found that even high concentrations of tetracycline can be completely degraded after 4 h of an electrochemical reaction.

In this paper, a Ti/Ta_2_O_5_-IrO_2_ anode was used to degrade TC. The IrO_2_ and Ti substrate bonding ability was strong and did not easily fall off and could be used in the acidic system for a long time stable. Ta_2_O_5_ was a chemically stable substance, and adding an appropriate amount of Ta_2_O_5_ to IrO_2_ coating not only can make the coating and titanium substrate contact parts become stable but also can play a role in the oxygen precipitation process to protect IrO_2_ particles from electrolytic consumption [20,21]. We have analyzed the structure and composition of the electrodes using the scanning electron microscope (SEM), the energy dispersive spectrometer (EDS) and the X-ray diffraction (XRD). The electrodes were tested using the linear sweep voltammetry (LSV) and the cyclic voltammetry (CV). The degradation effect of current intensity, the initial pH value, the electrode spacing, the initial TC concentration and their degradation kinetics were investigated. The degradation mechanism was analyzed by total organic carbon (TOC), UV–vis and identification of intermediates.

## 2. Materials and Methods

### 2.1. Experimental Materials

Tetracycline (C_22_H_25_N_2_O_8_Cl, USP) was purchased from Suzhou Shuertai Industrial Technology Co., Ltd. (Suzhou, China). CH_3_CN (HPLC grade) was supplied by Osenberg Chemicals, Sweden. Other reagents including ammonium acetate (Damao, Tianjin, China), trimethylamine (Damao, Tianjin, China), CH_3_COOH (Damao, Tianjin, China), Na_2_SO_4_ (Guangcheng, Tianjin), NaOH (Damao, Tianjin, China), H_2_SO_4_ (Damao, Tianjin, China) and EDTA (Maclean, Shanghai, China) were all AR grade and used without further purification. All solutions were prepared using deionized water with the resistivity near to 18.2 MΩ cm.

The Ti/Ta_2_O_5_-IrO_2_ anode (double-sided coating) and the Ti cathode were provided by Suzhou Shuertai Industrial Technology Co., Ltd. (Suzhou, China). The Ti/IrO_2_-Ta_2_O_5_ anode was prepared by spraying a Ir–Ta oxide solution on the surface of a titanium plate. The detailed process of preparing the Ti/IrO_2_-Ta_2_O_5_ anode was introduced in detail by Xu et al. [22]. Their sizes were 50 mm × 100 mm × 1 mm.

### 2.2. Experimental Methods

Electrochemical experiments were conducted in a 0.5 L electrolytic cell, made of quartz glass and stirred with a magnetic stirrer. The experiments were performed at 25 ± 2 °C, using 0.40 L of the 40 mg/L TC solution in 8.0 g/L of Na_2_SO_4_. The following experimental variables (and their ranges) were investigated: current intensity (2.5, 5, 7.5 and 10 mA/cm^2^), electrode spacing (20, 30, 40 and 50 mm), initial TC concentration (20, 40, 60 and 80 mg/L) and initial solution pH (4.74, 6.57, 7.78 and 9.48). The initial pH values were adjusted by aqueous NaOH and H_2_SO_4_. During the experiments, samples were drawn at certain time intervals for TC concentration measurement. They were filtered through a 0.22 μL syringe filter and stored in a 20 mL sampling tube at 5 °C for analysis.

### 2.3. Analytical Methods

The surface morphology of the Ti/IrO_2_-Ta_2_O_5_ electrodes and the structural morphology between the components were evaluated by scanning electron microscopy (SEM, Zeiss Sigma 300, Zeiss, Germany), X-ray energy spectroscopy (EDS, K alpha, ThermoFisher Scientific, Waltham, MA, USA) and X-ray diffractometry (XRD, D-12489, Bruker, Germany). An electrochemical workstation (CHI 660C, Chinstruments, Shanghai, China) was used to perform LSV and CV tests in a 0.1 M Na_2_SO_4_ electrolyte. The working electrode was a Ti/IrO_2_-Ta_2_O_5_ electrode (1 mm × 50 mm × 100 mm), the reference electrode was a saturated Ag-AgCl electrode and the counter electrode was a 1.0 cm × 1.0 cm platinum sheet electrode. Intermediates were detected by a high-performance liquid chromatography-mass spectrometry (HPLC-MS, Q Exactive Focus, ThermoFisher Scientific, Waltham, MA, USA). Total organic carbon (TOC) was measured with a TOC analyzer (TOC-L CPH, Shimadzu, Japan). The ultraviolet–visible absorption spectra (UV–vis) were measured by a UV spectrophotometer (TU-1900, PersinERSIE, Beijing, China).

The concentration of TC was determined with a high-performance liquid chromatography (HPLC, LC-10AT, Shimadzu, Japan), equipped with a Wondasil™ C-18 column (4.6 mm × 150 mm, 5 µm). A mixture of acetonitrile and ammonium acetate solution with the ratio of 17:83 (*v*/*v*) was used as the mobile phase. The ammonium acetate solution was made up of ammonium acetate solution (0.15 mol/L), EDTA solution (0.01 mol/L) and trimethylamine with the proportion of 100:10:1, and the pH value was adjusted to 8.5 by CH_3_COOH, as described in Chinese pharmacopoeia 2015. The elution was performed at a flow rate of 1.0 mL min^−1^, a wavelength of 275 nm, an injection volume of 20 μL and a column temperature of 25 ± 2 °C.

The intermediates were detected by the HPLC interfaced with a tandem mass spectrometer (LCMS-8040, Shimadzu, Shimane, Japan) with an InertSustain C18 column (4.6 mm × 150 mm, 5 µm). The column temperature was 40 °C. The mobile phase was a mixture of acetonitrile and deionized water at a ratio of 1:1 (*v*/*v*), a flow rate of 0.2 mL/min and an injection volume of 5 μL. The MS conditions were in positive ion detection mode. The following parameters were used: ion source temperature 523 K, dry gas flow rate 15.0 L/min.

## 3. Results

### 3.1. Electrode Morphology and Electrochemical Analysis

**Figure 1:**
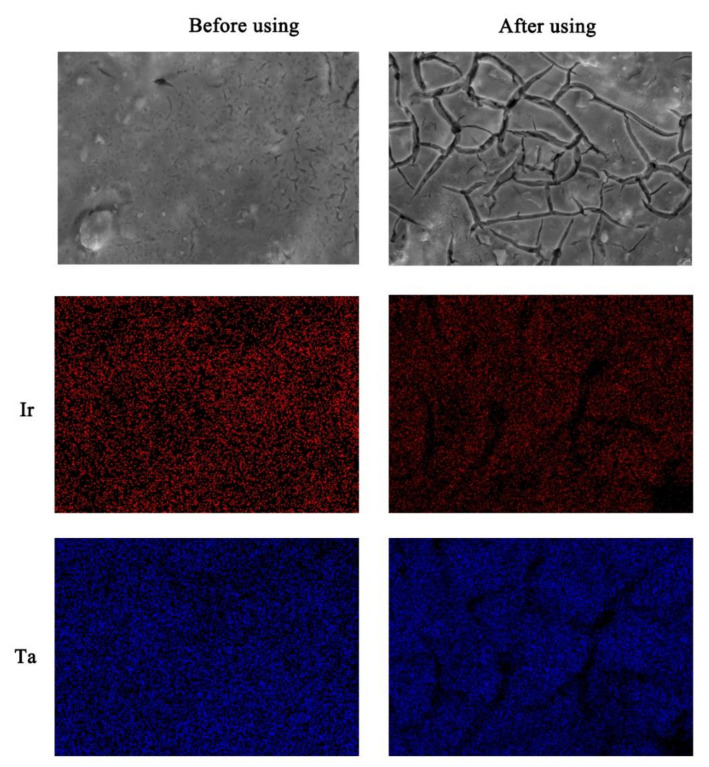
SEM and EDS patterns of the Ti/Ta_2_O_5_-IrO_2_ electrode.

Figure 1 shows SEM images (5000 magnification) and EDS images of the Ti/Ta_2_O_5_-IrO_2_ electrode. The Ti/Ta_2_O_5_-IrO_2_ electrode is sprayed with gold, and the acceleration voltage is 15.00 kv. Before use, the electrode surface was dense and the Ti/Ta_2_O_5_-IrO_2_ coating was able to cover the Ti substrate uniformly. The element Ir reduces cracking and allows the coating to bond more strongly to the substrate, thus giving the electrode a dense surface structure [23]. This structure not only prevents contaminants from penetrating into the Ti substrate through the oxide coating, but also effectively prevents the contact of highly valent metal oxides generated during electrolysis with the Ti substrate. The service life, catalytic activity and stability of the electrode can be improved. After 300 h of use, a cracked structure appeared on the surface of the electrode. As can be seen from the EDS pattern, the areas where the cracks were produced did not show the presence of the elements Ir and Ta. This inevitably reduces the catalytic activity and service life of the electrode and reduces the stability of the electrode.

**Figure 2:**
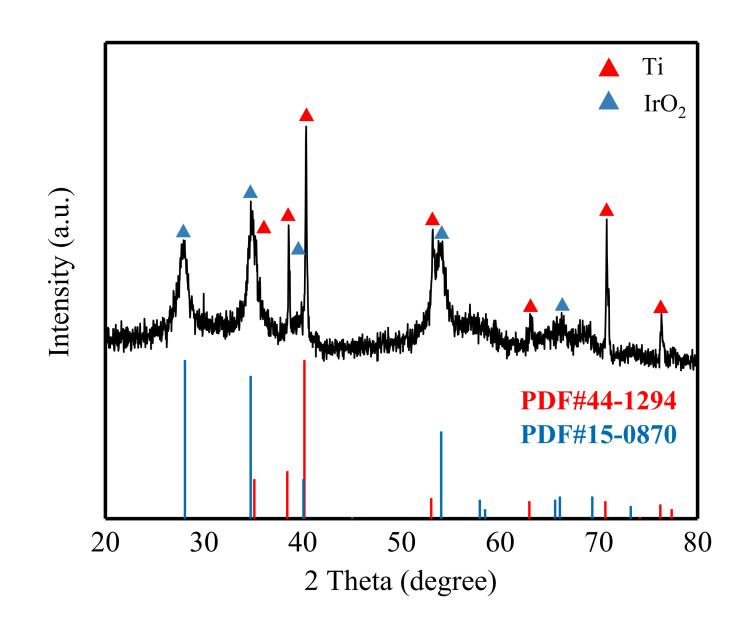
XRD pattern of the Ti/Ta_2_O_5_-IrO_2_ electrode.

Figure 2 shows the XRD pattern of the Ti/Ta_2_O_5_-IrO_2_ electrode. XRD diffraction angle range is 20–80°. As can be seen in Figure 2, the Ti/Ta_2_O_5_-IrO_2_ electrode has sharp peaks and high peak intensities for each of the diffraction peaks, indicating good crystallinity of the sample. The main diffraction peaks are located at 2θ = 27.92°, 34.73°, 38.57°, 40.34°, 53.18°, 54.14°, 70.79° and 76.34°. The diffraction patterns are in agreement with the standard cards Ti (PDF#44-1294) and IrO_2_ (PDF#15-0870), indicating that the above diffraction peaks are those of Ti and IrO_2_. Through the analysis of Jade software, it was found that the average half-width of a Ti crystal was 0.1424, and the average half-width of IrO_2_ is 0.512. The diffraction peaks of Ti are sharp and those of IrO_2_ are relatively broad. According to Scherrer’s formula, the average particle size of a Ti crystal was 14.05, and the average particle size of IrO_2_ was 3.13. It can be concluded that Ti has a larger crystal size and IrO_2_ has a smaller crystal size and crystallizes well.

**Figure 3:**
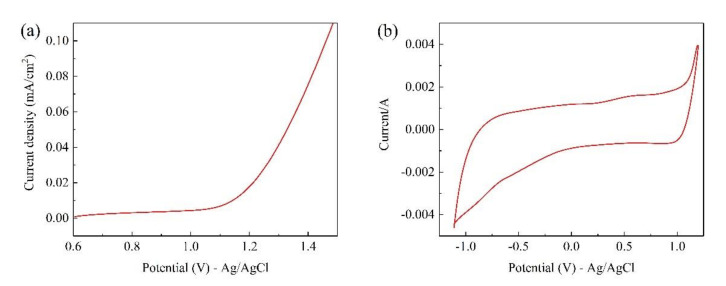
LSV (**a**) and CV (**b**) plots for Ti/Ta_2_O_5_-IrO_2_.

Figure 3 shows the LSV image and CV image of the Ti/Ta_2_O_5_-IrO_2_ electrode. Tests were carried out at a scan rate of 10 mV/s in 0.1 mol/L Na_2_SO_4_ solution. As can be seen from the image, the oxygen precipitation potential of the Ti/Ta_2_O_5_-IrO_2_ electrode is 1.08 V. The lower oxygen precipitation potential reduces the energy consumption of the reaction. Ti/Ta_2_O_5_-IrO_2_ electrodes not only have a dense surface structure, but also possess a low oxygen precipitation potential. It has good performance in electrochemical catalytic reactions.

### 3.2. Factors Influencing TC Electrochemical Oxidation

**Figure 4:**
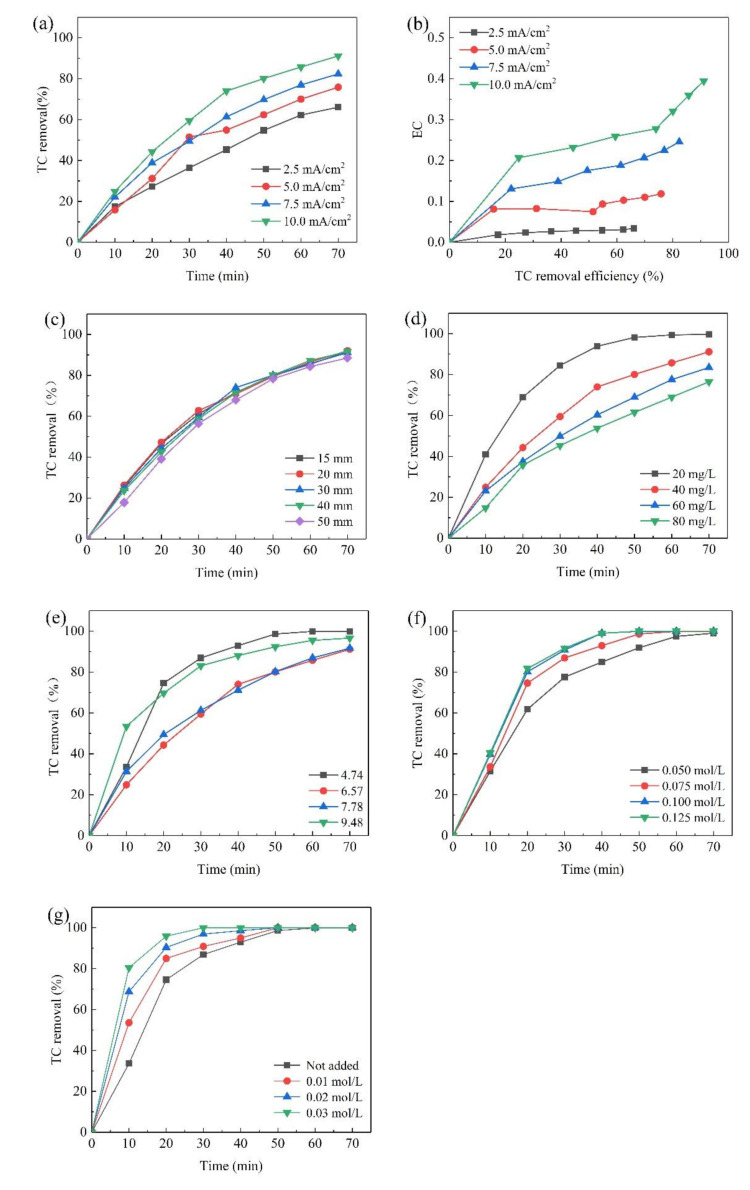
Effects of current density (**a**,**b**), electrode spacing (**c**), initial concentration (**d**), solution pH (**e**), electrolyte concentration (**f**) and Cl^−^ concentration (**g**) on TC degradation.

The increase in current density could effectively promote the generation and transfer of electrons. Within a certain range, the efficiency of catalytic degradation of organic matter will increase with the increase in current density. However, continuing to increase the current density will increase the intensity of side reactions and affect the catalytic degradation process. Figure 4a showed the effect of current density on TC removal and reaction rate constant (Ka). The removal of TC was accelerated by increasing the current density. At the same time, the degradation kinetics analysis was depicted in Table 1. Good linear fit plots were obtained, suggesting that the degradation of TC followed the pseudo first-order kinetic mode. An increase in the current density led to an increase in reaction rate constant (Ka) values from 0.01554 min^−1^ to 0.03426 min^−1^ within 70 min electrolysis. Current density was a primary parameter to determine the generation rate of hydroxyl radicals (·OH) on the electrode surface and the energy consumption during the electrochemical treatment. The high current density was helpful to produce a larger amount of the ·OH available for electrochemical degradation of TC. When the concentration of generated ·OH is too high, it is difficult to react with the organic matter around the electrode in a short period of time, so its own self-quenching reaction occurs to generate O_2_, reducing the utilization rate of the ·OH, while the cathode surface gains more electrons, promoting the four-electron reduction of O_2_ to generate an H_2_O reaction, reducing the two-electron reduction to generate an H_2_O_2_ reaction, thus reducing the concentration of H_2_O_2_ in the solution, which will reduce the rate of oxidative decomposition of organic matter. Moreover, the electron transfer was limited by the concentration of electrolyte in the solution. As the current density increased, the degradation rate did not increase significantly. This was verified by experiments by Wang [12]. Increasing current density caused the increase in energy consumption (EC) (shown in Figure 4b). Especially, as the removal ratio exceeded 80%, EC would dramatically increase, indicating that under the low concentration, TC degradation was no longer controlled by current density, and more energy was consumed by the side reaction or converted into heat [22,24]. During the process of TC degradation by the electrochemical method, the pursuit of a high degradation rate would lead to a rapid increase in EC. Thus, it was uneconomical to pursue a high degradation efficiency of TC only by increasing current density. In this system, an 80% degradation rate was more appropriate.

The smaller the electrode spacing, the greater the force between the electrodes, and the better the degradation effect. If the distance between the plates is too small, short circuits are more likely to occur, and the anode surface is also prone to passivation, which increases energy consumption. Too large electrode spacing increases the resistance between the electrodes, and energy consumption also increases. The effects of electrode distance on TC removal were shown in Figure 4c. The variation of K values was also negligible with the range from 0.3191 min^−1^ to 0.3556 min^−1^ (Table 1). When electrode distance was 20 mm, the maximum removal was 92.02% after 70 min. When the distance between the plates was reduced to 15 mm, the degradation rate was reduced to 91.59%. When current passes through the electrode, various ions in the solution will move in a certain direction under the action of the electric field, and the phenomenon of “electromigration” will occur. Therefore, the diffusion and convection of solution components will occur. When the electrode spacing is increased, the diffusion and convection of the solution components are reduced, and the reaction rate of the organic matter inside the solution with the ·OH is slowed down because the ·OH is generated near the electrode, resulting in a lower TC degradation rate. Under certain conditions of the applied current, as the electrode spacing increases, the mass transfer resistance increases, leading to a decrease in removal rate; at the same time, the voltage between the electrodes increases and the side reactions increase. It was beneficial to choose a small electrode distance to maximize the removal rate and minimize energy consumption. However, a very small electrode distance was difficult to prevent short circuits, and also, the solution was difficult to diffuse in such a small area [12,15,22,24]. In this study, electrode spacing of 20 mm was considered the optimum.

The effects of the initial concentration of TC on electrochemical degradation were shown in Figure 4d. TC was almost completely degraded at 70 min when the initial concentration was 20 mg/L, suggesting that the speed of the ·OH production was faster than that of the TC diffusion to the electrode surface [23,25]. Thus, the TC degradation on a Ti/Ta_2_O_5_-IrO_2_ anode was a diffusion-controlled electrochemical process, which was in agreement with Zhao et al. [26,27]. Due to the same galactic conditions, the direct or indirect oxidation processes produced similar concentrations of the electron transfer and reactive groups. At low initial concentrations, less intermediates were produced and the TC reaching the electrode surface could be completely degraded. With the increase in initial TC concentration, more and more TC and produced intermediates cover the electrode surface, preventing more TC and intermediates from reacting with the OH radicals and consuming a significant portion of the ·OH radicals in the process, leading to a gradual decrease in removal rates [16,24,25,28,29]. The simulated Ka values decreased from 0.08656 min^−1^ to 0.02009 min^−1^ for initial concentrations from 20 mg/L to 80 mg/L. This effect of initial TC concentration on Ka values was consistent with Qian et al., who used a Ti/SnO_2_-Sb_2_O_3_/PbO_2_ anode to investigate the degradation kinetics of TC [23,25]. The Ka values dramatically reduced when the initial concentrations increased from 20 mg/L to 40 mg/L, but slowly decreased when the concentrations varied from 60 mg L^−1^ to 80 mg L^−1^, indicating that the intermediates reaching the electrode surface and competing with TC within this concentration range might gradually reach a saturation state. Even if the TC initial concentration was further increased, the removal rate of TC would not be significantly affected.

During the electrochemical process, initial solution pH affects not only the formation of free radicals, but also the existing state of organic matter in the solution. Thus, it is considered as one of the most important factors affecting the degradation process [27,28,30,31]. As can be seen in Figure 4e, the optimum pH value was 4.74 and the Ka value was about 2 times higher than that at pH 6.57 and pH 7.78. After 70 min of electrolysis, the removal ratio reached 99.86%, which was much higher than those at the other three pH values. Acidity condition was thought to facilitate free radical formation, which had a positive effect on TC oxidation [29,32]. The oxidation potential of hydroxyl radicals in acidic conditions (+2.85 V) was much higher than that in alkaline conditions (+2.02 V), and the high potential level could enhance TC degradation. Furthermore, oxygen precipitation was inhibited under acidic conditions, improving the efficiency of TC oxidation. Under alkaline conditions, the large amount of the OH^−^ contained in the solution can react with the large amount of the ·OH catalyzed by the electrode to produce the less oxidizing ·O^−^ [30,33]. Its present species in solution is strongly affected by the pH value due to the protonation or deprotonation of amino and hydroxyl groups bound to the aromatic ring [31,32,34,35]. Under different pH conditions, TC could be dissociated into different species, such as protonated form TCH^3+^ (pH < 3.3), neutral form TCH_2_ (3.3 < pH < 7.7) and deprotonated form TCH^−^ (7.7 < pH < 9.7) and TC^2−^ (pH > 9.7) [33,36]. Due to electrostatic attraction, the negative TCH^−^ was easier to reach the anode for degradation reaction. Thus, at an initial solution pH of 9.48, the degradation rate of TC was higher than those at pH 6.57 and 7.78 but lower than that at pH 4.74.

During the catalytic oxidation process, the electrolyte concentration will affect the concentration of free ions in the solution, thereby affecting the conductivity of the electrolyte. The higher the electrolyte concentration, the faster the electron transfer rate. However, while the electrolyte acts as an electron transfer carrier, a part of it will also react with the oxidizing active groups in the solution, reducing the utilization of electrons and causing a decrease in the reaction rate. As seen in Figure 4f, the best degradation ratio of 98.99% was achieved when Na_2_SO_4_ was 0.125 mol/L. The first-order kinetic analysis was performed on the concentration change of TC within 40 min, as shown in Table 1. In a specific range, increasing the electrolyte concentration was beneficial to the degradation of TC. However, when the electrolyte concentration was too high (0.100 mol/L), the degradation rate of TC did not increase significantly. Too low electrolyte concentration would affect the electron transfer between electrodes, and additional electrode voltage was needed, increasing the energy consumption of the reaction. During the process of the electron transfer, persulfate and sulfate radicals would be generated, reducing the concentration of the ·OH [37]. The pathways of by-product generation were shown in Equations (1) and (2).

2SO_4_^2−^→S_2_O_8_^2−^ + 2e^−^ (1)

S_2_O_8_^2−^ + e^−^→SO_4_^2−^ +·SO_4_^−^ (2)

Electrolytes produce substances with oxidizing activity in the presence of electric current, thus promoting or inhibiting the degradation of organic matter [38]. To investigate the effect of Cl^−^ concentration on the degradation effect of TC, experiments were conducted by setting Cl^−^ concentrations of 0.01, 0.02 and 0.03 mol/L. The change of TC degradation rate is shown in Figure 4f. The first-order kinetic analysis was performed on the concentration change of TC within 40 min, as shown in Table 1. From Figure 4f, when the chloride ion concentration was 0.03 mol/L, the TC had been completely degraded after 30 min of reaction. It could be seen that the addition of Cl^−^ promoted the catalytic degradation of CAP. The experiment of Frontistis et al. also verified this [18]. Reactive chlorine, having a powerful oxidative destructive ability for organic compounds, was generated during electrolysis [39]. The reactions are shown in Equations (3)–(5):

2Cl^−^→Cl_2_ + 2e^−^ (3)

Cl_2_ + H_2_O→HClO + Cl^−^ + H^+^ (4)

HClO→ClO^−^ + H^+^ (5)

The high current density made it easier to obtain a high TC degradation rate. However, too high current density would reduce current utilization. Therefore, in our experiment, the current density of 10 mA/cm^2^ was the most reasonable. Through our experiments, it was found that the electrode spacing has little effect on the degradation of TC. It would be affected by electrolyte concentration and other factors. The effect of the initial concentration of TC on the degradation rate of TC was obvious. The lower TC initial concentration made it easier to obtain a higher TC degradation rate. However, it was not advisable to pursue a high degradation rate and ignore the amount of TC that is degraded at the same time. While the pH value affected the production of active groups, it also affected the morphological stability of the organic matter. It was found from experiments that low pH value made it easier to obtain a high TC degradation rate. Increasing the electrolyte concentration is beneficial to TC degradation, but too high electrolyte concentration does not significantly increase the TC degradation rate. Cl^−^ will generate strong oxidizing active groups under the action of electrons. Adding Cl^−^ was easier to promote the degradation of TC.

### 3.3. Electrochemical Oxidation Mechanism of TC

#### 3.3.1. UV–Vis Adsorption Spectra

**Figure 5:**
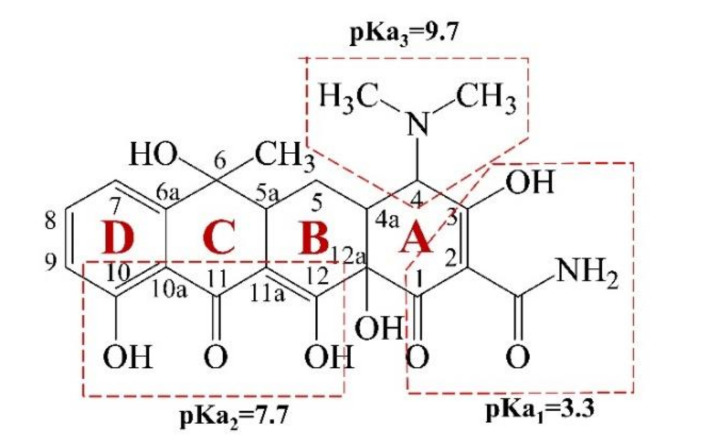
Chemical structures and pKa values of TC.

**Figure 6:**
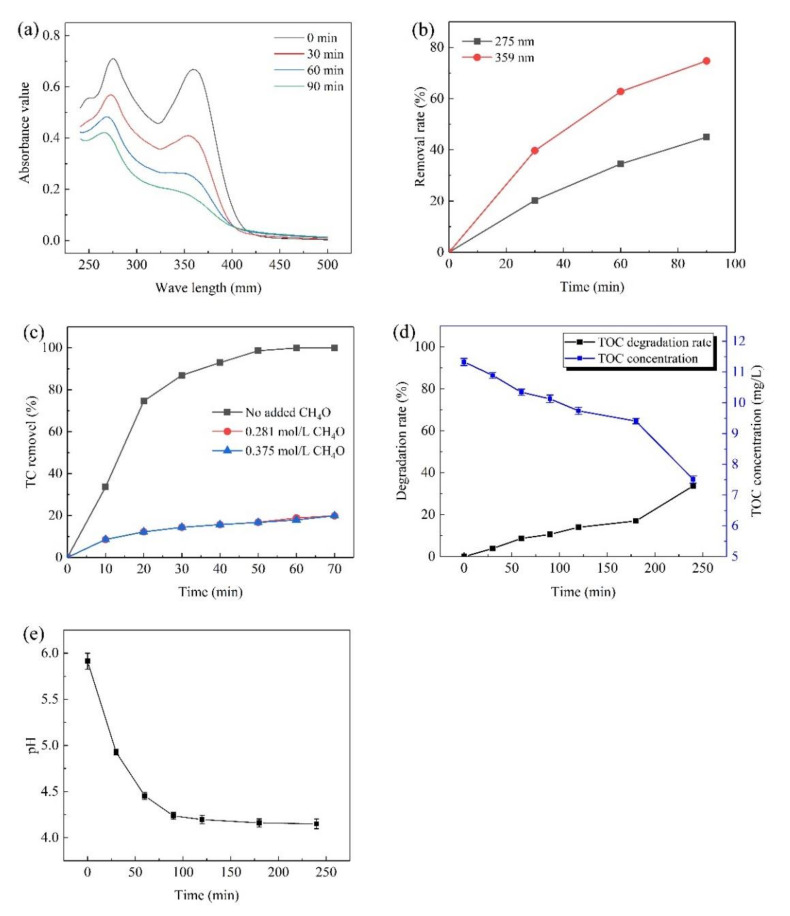
Changes in UV–vis absorption spectra (**a**), the relative absorbance removal at 275 nm and 359 nm (**b**), the ·OH verification (**c**), the TOC (**d**) and pH (**e**) during electrochemical degradation.

Two main absorption peaks were observed at approximately 275 nm and 359 nm before treatment. Wessels et al. indicated that the 275 nm adsorption peak was associated with the ring A structure, including the amide group, ketone group and enolic hydroxyl group, and as shown in Figure 5, the chromophores on the rings of B, C and D contributed to the both absorption peaks [34,40]. The UV–vis adsorption spectra from 240 nm to 500 nm at different time were applied to clarify the structural changes during the TC degradation process (Figure 6a).

The variation of relative absorbance removal with time was plotted in Figure 6b. The relative removals at 359 nm were always higher than those at 275 nm. A similar result was also reported by other research. The faster decrease at 359 nm might be due to the destruction of the B, C and D aromatic rings conjugate structure under an· OH attack. The slower decrease at 275 nm might be due to a slower degradation of the A ring or the formation of some intermediates that adsorbed at 275 nm [35,36,41,42]. While Dong et al. obtained a different conclusion that attenuation apparently increased at 275 nm absorption peaks, and slightly decreased in the 359 nm absorption regions, when the effect of zero-valent iron (ZVI) on TC degradation was investigated. They attributed this difference to a more likely combination for ZVI with the A ring and a weaker effect on the BCD rings [37,43].

#### 3.3.2. Detection of Hydroxyl Radicals

Because the residence time of the ·OH in the solution is concise, direct measurement is difficult to operate. The free radicals generated during the degradation process were verified by adding a free radical scavenger. CH_4_O is used as an ·OH capture agent, and the reaction rate with the·OH is 9.7 × 10^8^ M^−1^·s^−1^ [44]. In the TC solution, 0.281 mol/L and 0.375 mol/L of CH_4_O were added, respectively, and the experiment was carried out. The experimental results were shown in Figure 6c. As can be seen from the graph, the addition of CH_4_O significantly reduced the degradation rate of TC. After 70 min, the degradation rates were reduced by 79.99% and 79.98%, respectively. The added CH_4_O can react rapidly with the ·OH. The results indicate that a strong oxidizing ·OH was generated in the electrochemically catalyzed reaction, which degrades the TC. The TC degradation rates after the addition of 0.281 mol/L and 0.375 mol/L of CH_4_O did not change significantly, indicating that the addition of CH_4_O was excessive. This indicated that in the catalytic reaction, the OH will be produced to degrade TC.

#### 3.3.3. TOC

TC removal reached more than 90% after 90 min, while there were few changes in the TOC content, indicating that in this electrochemical system, TC might be converted into the more oxidized intermediates, which were recalcitrant to the oxidants generated electrochemically or were stable when exposed to direct electrochemical oxidation at the anode surface. These results were consistent with that of Giraldo et al., who suggested that the poor mineralization of oxacillin at Ti/IrO_2_ anode in the presence of NaCl as support electrolyte was attributed to the formation of the refractory organic compound that no longer reacted with the chlorinated oxidative agents [12,45]. Thus, the nature of the intermediates determined whether they were completely mineralized or partially degraded. As the reaction progresses to 180 min, the TOC concentration drops rapidly, indicating that the intermediate product has started to be degraded. At 240 min into the reaction, the TOC degradation rate was 33.69%.

Panizza et al. attributed the low TOC removal to reaction with the selective, partial conversion of organic compounds by the reaction with chemisorbed active oxygen at a DSA anode. The chemisorbed active oxygen was formed by the adsorbed hydroxyl radicals to the lattice of an oxide anode (shown in Equations (6)–(8)) [38,46].

IrO_2_ + H_2_O^®^IrO_2_(·OH) + H^+^ + e (6)

IrO_2_(·OH) ^®^IrO_3_ + H^+^ + e (7)

TC + IrO_3_^®^IrO_2_ + intermediates (8)

Also, the DSA anode had low oxygen precipitation overpotential. During the electrochemical process, it was favorite for the secondary reaction of oxygen precipitation in comparison with organic oxidation (Equation (9)).

2H_2_O^®^O_2_ + 4H^+^ + 4e (9)

The variation in the pH value (Figure 6d) showed that the pH gradually decreased from 5.93 to 4.22 during the 4 h electrochemical process. As documented previously, the decrease in pH values was probably due to the production of protons, as shown in Equations (6), (7) and (9).

#### 3.3.4. Identification of Intermediates and the Proposed Degradation Pathways

**Figure 7:**
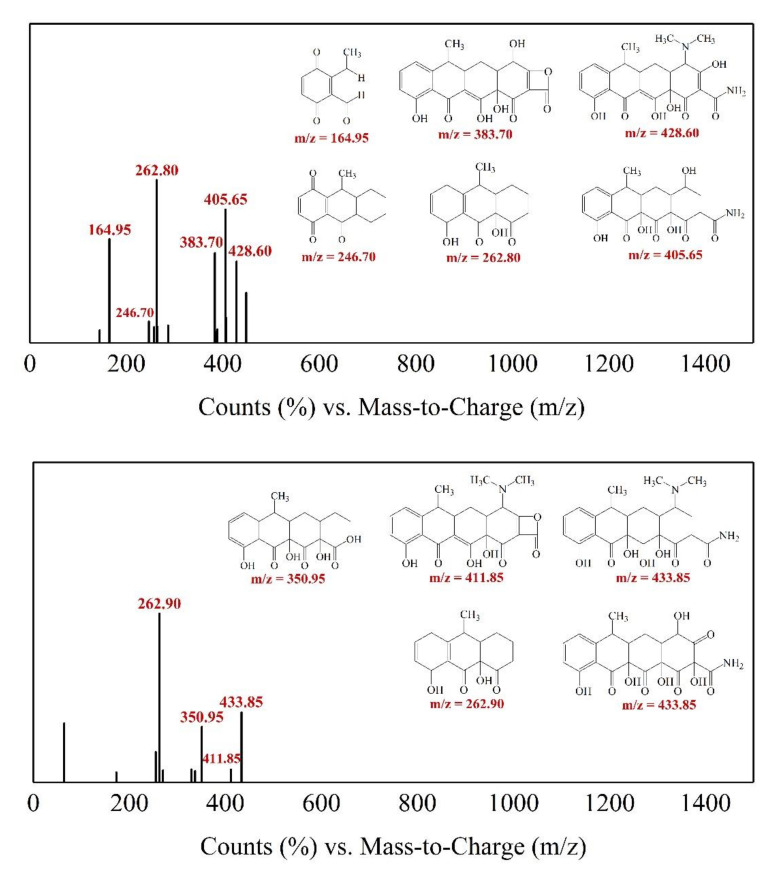
Degradation of tetracycline after a reaction time of 90 min in aqueous media was monitored by total ion chromatography using HPLC-MS. The inset shows the mass spectra of the eluted products at 1.782 min, 1.890 min.

**Figure 8:**
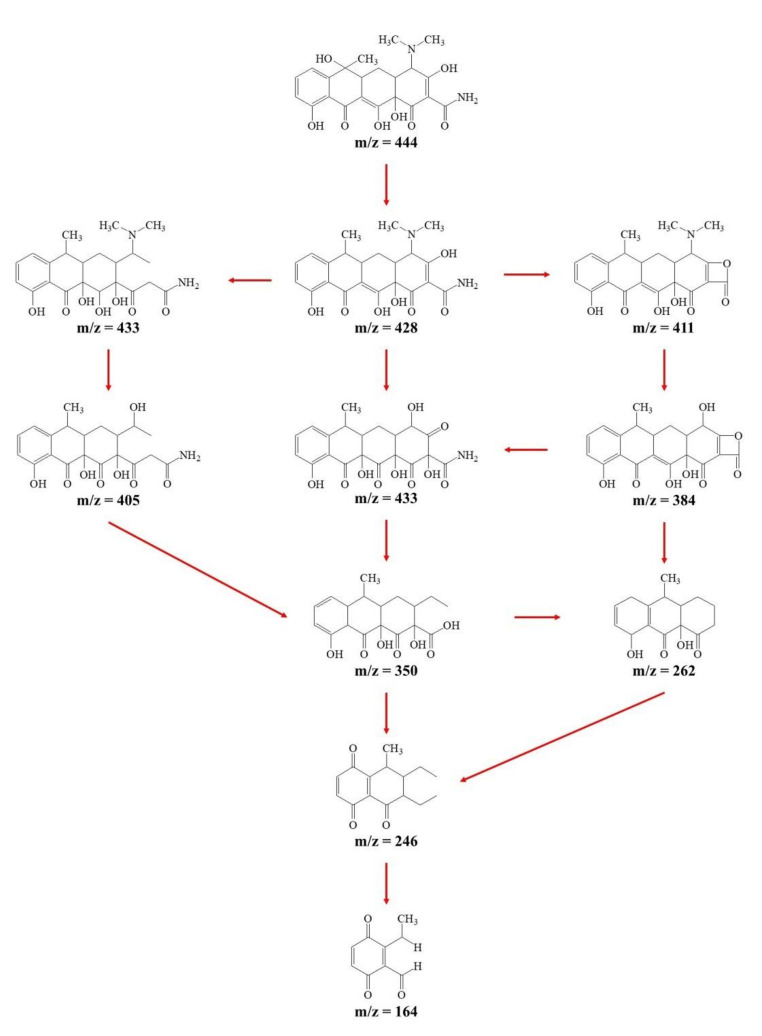
Proposed pathways of TC degradation by Ti/Ta_2_O_5_-IrO_2_ anode.

HPLC-MS was employed to identify the produced intermediates of TC at pH 6.57 for 90 min degradation. For TC, a *m*/*z* 444 molecular was observed, and nine intermediates with *m*/*z* values of 433, 428, 411, 405, 383, 350, 262, 246 and 164 were detected (shown in Figure 7). Their structures and degradation pathways could be inferred (shown in Figure 8).

First, water loss was proposed to form the product *m*/*z* = 433, due to the oxidation of the hydroxyl group bound at C6 atom, which was consistent with the results reported by Dalmázio et al. [36,42] and Wang et al. [39,47]. In the proceeding degradation, the ·OH would react with electron-rich functional groups. Therefore, the six potential reactive sites might compete for oxidation: double bonds (C2–C3 and C11a–C12), an amido group (C2), dimethylamino (C4) and phenolic hydroxyl groups (C7 and C9) [40,41,42,48,49,50]. For *m*/*z* = 428, there might be three subsequent degradation pathways. The first pathway: the C11a-C12 double bond was more susceptible to free radical attack and generated hydroxyl and keto groups [42]. After the C11a–C12 double bond was broken, the ·OH continued to attack the double bond at C2–C3, causing the A ring to break at C3, and at the same time, the chemical bond between C3 and the hydroxyl group would be broken to obtain a compound with *m*/*z* = 433. The second pathway was that the ·OH attacked the dimethylamino group at C4, and another compound with *m*/*z* = 433 would be obtained by adding C2–C3 double bond [43,51]. The third pathway was that the ·OH attacked the amide group at C2 and the hydroxy group at C3 and removed an NH_3_ molecule to obtain a compound with *m*/*z* = 411. Next, the first compound with *m*/*z* = 433 and the compound with *m*/*z* = 411 would undergo the hydroxyl substitution reaction of the dimethylamino group at C4 to obtain two compounds with *m*/*z* = 405 and *m*/*z* = 383 [40,41,44,48,49,52]. With a further reaction, the ·OH continuously attacked the D ring, attacking the ortho (C9) or para (C7) position of the phenolic hydroxyl group and the phenol ring. Compared with the ortho position, due to the function of the C6 functional group, the para reactivity was stronger. Therefore, a carbonyl group was formed at C7 and C10 [45,46,53,54]. With further reactions, the A, B and C rings were successively broken to obtain compounds with *m*/*z* = 350, *m*/*z* = 262, *m*/*z* = 246 and *m*/*z* = 164.

## 4. Conclusions

The Ti/Ta_2_O_5_-IrO_2_ electrode has a dense surface structure and the elements Ir and Ta are uniformly doped on the electrode surface. With a low oxygen precipitation potential, the energy consumption of the reaction can be reduced. Electrochemical degradation can effectively remove TC by using a Ti/Ta_2_O_5_-IrO_2_ anode. LSV and CV tests show that the electrode has a low oxygen precipitation potential and low energy consumption in the catalytic process. Under the experimental conditions, the degradation process followed the first-order kinetic model. The removal efficiency of TC and the EC increased with the increase in current density. It was uneconomical to pursuit high removal efficiency only by increasing current density. Reducing the initial TC concentration and electrode spacing and adding Cl^−^ could increase the degradation efficiency of TC. The highest degradation efficiency was obtained at pH 4.74 during the range of 4.74–9.48 and its Ka value was more than two times higher than those at other pH values. The relative decreases at 359 nm were always higher than those at 275 nm, which was due to the destruction of the B, C and D aromatic rings conjugate structure, a slower degradation of A ring or the formation of some intermediates that adsorbed at 275 nm. There were few changes on the TOC concentration using a Ti/Ta_2_O_5_-IrO_2_ anode during the degradation process of 1.5 h, while the pH values decreased from 5.93 to 4.22. Nine major intermediates were identified by HPLC-MS and three degradation pathways were proposed.

## Figures and Tables

**Table 1:** Efficiency and kinetics of TC electrochemical degradation.

Parameters		Degradation Efficiency (%)	Rate Constants (ka, min^−1^)	R^2^
Current density (mA/cm^2^)	2.5	66.08	0.01554	0.996
	5	75.84	0.02022	0.989
	7.5	82.33	0.02453	0.998
	10	91.12	0.03426	0.995
Electrode spacing (mm)	15	91.59	0.03420	0.992
	20	92.02	0.03484	0.989
	30	91.12	0.03426	0.995
	40	91.62	0.03456	0.992
	50	88.49	0.03191	0.995
Initial TC concentration (mg/L)	20	99.69	0.08656	0.982
	40	91.12	0.03426	0.995
	60	83.46	0.02510	0.993
	80	76.45	0.02009	0.994
Initial pH	4.74	99.88	0.10420	0.936
	6.57	91.12	0.03426	0.995
	7.78	91.68	0.03420	0.992
	9.48	96.57	0.04370	0.988
Electrolyte concentration (mol/L)	0.050	84.89	0.04895	0.993
	0.075	92.90	0.06914	0.985
	0.100	98.97	0.11025	0.915
	0.125	98.99	0.20280	0.839
Cl^−^ concentration (mol/L)	0	92.90	0.06914	0.985
	0.01	94.99	0.07614	0.974
	0.02	98.56	0.10789	0.992
	0.03	99.99	0.25998	0.877
